# Causes of Death during the Intravenous Infusion of Dimethylsulphoxide and Hydrogen Peroxide in the Course of Alternative Medicine Therapy

**DOI:** 10.3390/toxics11080652

**Published:** 2023-07-28

**Authors:** Szymon Rzepczyk, Paweł Świderski, Karina Sommerfeld-Klatta, Artur Tezyk, Magdalena Łukasik-Głębocka, Barbara Zielińska-Psuja, Zbigniew Żaba, Czesław Żaba

**Affiliations:** 1 Department of Forensic Medicine, Poznan University of Medical Sciences, Rokietnicka 10, 60-806 Poznan, Poland; 2 Department of Toxicology, Poznan University of Medical Sciences, Dojazd 30, 60-631 Poznan, Poland; 3 Department of Emergency Medicine, Poznan University of Medical Sciences, Rokietnicka 7, 60-806 Poznan, Poland

**Keywords:** DMSO, hydrogen peroxide, intoxication, alternative medicine, medication error

## Abstract

Unconventional (alternative, natural) medicine in Poland and worldwide includes hundreds of non-scientifically verified “treatment” modalities. Among the most popular are biological therapies using chemical or natural compounds administered with injection or drip infusion. The latter has found the most excellent use in treating rheumatological and dermatological diseases and certain types of cancer. Vitamin infusions, curcumin, glutathione, perhydrol and dimethylsulphoxide (DMSO) have gained popularity among clients of natural medicine clinics. The present study aims to analyse the case of a 37-year-old woman who was administered infusions containing perhydrol and DMSO (0.5 mL 0.04% hydrogen peroxide/0.5 mL p.d.a DMSO in saline) due to a MTHFR A1298C mutation. After having the next infusion, the woman complained of nausea and then became unconscious. Subsequently, she suffered respiratory and cardiac arrest. Adequate resuscitation was undertaken. After being taken to the hospital, the patient was in critical condition and died due to increasing multiple-organ failure. Initially, there was suspected DMSO poisoning as it was the only compound to have been administered as an intravenous infusion. However, it was not until the analysis of the secured evidence that it became clear that the patient had also been given an intravenous solution of hydrogen peroxide, H_2_O_2_, and that there had been a mistake in preparing the intravenous perhydrol solution. The autopsy concluded that the immediate cause of death was an acute cardiopulmonary failure due to the toxic effects of intravenously administered hydrogen peroxide. This conclusion was established after the toxicological testing of the evidence and biological material secured during the patient’s treatment and autopsy. Products containing DMSO and perhydrol are not included in the lists of medicinal/therapeutical forms and preparations and thus are not authorised for marketing in Poland. In the case of perhydrol, apart from the topical use of diluted preparations for washing and cleansing wounds, no data on therapeutic use exist in the available scientific literature. Furthermore, “DMSO and perhydrol therapy” cannot even be considered a placebo effect, as both are toxic compounds which could, at most, cause poisoning symptoms rather than improve health.

## 1. Introduction

In recent decades, the number of alternative medicine providers and patients using their services has significantly increased [1,2,3,4,5,6]. In addition to marketing efforts and increased availability, a loss of trust in conventional medicine and medicines and the illusion of patient autonomy may explain the greater interest in such services [1,4,7]. Alternative medicine centres offer patients therapies that are often not in line with Evidence-Based Medicine but are marketed as tradition-based, effective and natural—which may indicate medical populism [4,7]. “Natural medicine clinics” are often run by people outside the health care system. They offer “detoxification” or “anti-cancer” treatments with intravenous infusions of very high, often toxic, doses of vitamins or reagents used in chemistry (e.g., concentrated DMSO or hydrogen peroxide), basing their methods on pseudo-scientific claims or disproven scientific concepts. Chronically ill people and cancer patients resort to alternative medicine services most readily [4]. There are disturbing reports of a much higher rejection rate of conventional medicine therapies by alternative practitioners and patients taking advantage of such methods and keeping them secret from their doctors [1]. The literature reports cases of severe injury or death due to faulty alternative medicine services by untrained practitioners [8,9,10,11,12,13,14,15]. In Polish law, so-called “alternative medicine therapy” is not explicitly prohibited, and investigations against such entities are initiated only in the event of exposing or causing damage to health or death. The aim of the study is to describe the diagnostic difficulties associated with post-mortem examination after an overdose of DMSO and hydrogen peroxide administered intravenously.

## 2. Case Report

### 2.1. Anamnesis and Circumstantial Data

A 37-year-old woman reported to a “natural medicine clinic” for another series of infusions with DMSO and hydrogen peroxide. The procedure was to be performed as a continuation of “oxygen therapy” due to the presence of the MTHFR A1298C mutation. The patient had no history of cancer in the past. The treatment was ordered to “cure the mutation”, according to the advice of an alternative therapist. All procedures were performed based on a wish and with agreement of the patient. The patient had been receiving the “therapy” for several months. Based on additional investigations at the “treatment” qualifying appointment, the patient was diagnosed with Graves–Basedow disease and gastrointestinal dysfunction due to oral hydrogen peroxide administration. In addition, the patient had previously used vitamin infusions and coconut oil as supportive treatments. A “natural medicine doctor” developed a “therapy” prescribing ten infusions over 3 months at intervals of several days with an accompanying vegetable diet. The preparations used in the therapy were 0.04% hydrogen peroxide in 250 mL of 0.9% NaCl and 10 mL of 90% DMSO in 250 mL of 0.9% NaCl. After 200 mL of the DMSO solution was administered, the patient developed severe dyspnoea, vomiting and a loss of consciousness. Emergency medical services were called. Based on the examination and history of the substances administered, the emergency medical team diagnosed pulseless electrical activity (PEA) due to DMSO poisoning (GCS 3, RTS 5). ROSC was achieved after 27 min of resuscitation. The patient required intubation and transport to an intensive care unit. The patient required adrenaline and fluid supplementation and an automatic cardiac massage machine during transport. After transfer to the intensive care unit, the patient’s condition was described as critical, with symptoms of increasing multi-organ failure due to an overdose of DMSO. A strong sulphurous odour from the patient could be smelled as well. Fluid therapy was continued, adrenaline and dobutamine infusion was connected and sedating midazolam and fentanyl were administered. After a toxicology consultation, a decision was made to deliver continuous renal replacement therapy (CRRT). In the following hours, the patient’s condition deteriorated despite treatment—multi-organ failure developed, as well as cerebral oedema and acute circulatory failure due to severe left ventricular systolic dysfunction. ALT (over 200 U/L), AST (over 200 U/L), GGTP (over 200 U/L) and troponin levels (over 14,000 ng/L) were significantly elevated. An ABG test showed profound acidosis (pH 7.065). An ultrasound examination and chest X-ray performed during hospitalization showed the presence of fluid in the pleural and peritoneal cavities. Despite intensive care, the patient died 24 h after admission.

### 2.2. Autopsy Findings

The police and the public prosecutor were notified of the case. The case was investigated, and an autopsy was ordered at the Department of Forensic Medicine. In addition, the containers and instruments used to administer the infusions at the “natural medicine clinic” were secured. The autopsy was performed 2 days after death, revealing increased cerebral oedema, more than 1000 mL of transudate fluid in the pleural cavities and the passive congestion of the internal organs. By the time the autopsy data provided by investigators suggested acute DMSO intoxication, hydrogen peroxide infusion was not mentioned. An air embolism specific test was performed with inconclusive results. The ventricles and atria of the heart contained liquid blood. Heart wall muscle thickness was within the normal range for sex and age. In addition, macroscopically, the kidneys, liver and spleen showed no changes indicative of toxic damage. The bile duct was patent. The entire length of the digestive tract was filled with digested food remnants. The uterus and ovaries were of a typical size for age, without visible pathologies. In addition, material was taken for histopathological examination, which confirmed cerebral oedema and showed features of early myocytolysis of the myocardial fibres and increased pulmonary oedema. In the course of the investigation and questioning of the “natural medicine clinic” staff, information was obtained about an error by one of the midwives/medical staff preparing and administering the preparations, supposedly involving the administration of a too high hydrogen peroxide dose, which was not communicated to the emergency medical team during the rescue of the patient. A toxicological analysis of the biological material (blood collected during hospitalization and blood and urine secured at autopsy) confirmed the presence of DMSO. A toxicological examination of the containers and instruments used to administer the intravenous preparations showed concentrations of hydrogen peroxide significantly higher than those recorded in the documentation (0.04%). 

### 2.3. Toxicological Investigations

Evidence was secured for the toxicological test in the form of two glass bottles labelled “DMSO Sterile” containing traces of colourless liquid (Nos. 1 and 2), a polyethene container intended for IV fluids labelled “Natrium Chloratum 0.9%” containing traces of colourless liquid (No. 3), a polyethene container intended for IV fluids with a stick-on label stating “Water for injections” containing approximately 130 mL of colourless liquid (No. 4), two polyethene containers intended for infusion liquids labelled “Natrium Chloratum 0.9%” and with the handwritten inscription “DMSO” (Nos. 5 and 6) and a polyethene container intended for infusion liquids labelled “Water for injection” with the handwritten inscription “H_2_O_2_” and the date of preparation of the mixture (No. 7). In addition, a 25 mL syringe containing approximately 15 mL of colourless liquid (No. 8) and IV fluid tubing with a dropper were secured. An analysis of the evidence was carried out using gas chromatography coupled with mass spectrometry (GC-MS). Liquid samples (Nos. 1 to 8) were analysed after dilution with acetonitrile and methanol to confirm the presence of dimethylsulphoxide. Evidence nos. 1, 2, 5 and 6 showed the presence of DMSO (Table 1). In evidence nos. 3, 4, 7 and 8, no chromatographic peaks were found to confirm the presence of this substance. Then, high-performance liquid chromatography coupled with tandem mass spectrometry (HPLC-MS/MS) was performed. Liquid samples (Nos. 1 to 8), after dilution with the mobile phase (0.1% formic acid solution in water supplemented with 5% acetonitrile), were analysed to confirm the presence of DMSO. DMSO was present in evidence nos. 1, 2, 5 and 6, whereas, as in the GC-MS analysis, no chromatographic peaks were found in evidence nos. 3, 4, 7 and 8, confirming the detection of the substance sought. A classical instrumental method was used to confirm the presence of hydrogen peroxide in the evidence materials, consisting of adding a 0.1 M H_2_SO_4_ and 0.02 M KMNO_4_ solution to the test specimen. The solution became colourless in samples 3, 7 and 8, and some gas evolved. This reaction is characteristic of the presence of hydrogen peroxide. In addition, a quantitative analysis was performed to determine the percentage of hydrogen peroxide. After the acidification of sample nos. 3, 7 and 8 with 0.1 M of sulphuric acid, titration with a 0.02 M KMNO4 solution was performed until a permanent raspberry colour was obtained. Water for injection and the 0.9% NaCl solution were used as a control. Applying the two references and the analytical instrumental methods gave rise to the results represented in Table 1.

Biological material taken from the victim was used to perform tests crucial for confirming the poisoning. Blood and urine samples collected during the autopsy and blood samples and skin with mucous membrane swabs taken during hospitalization were secured for the analysis. Skin and mucous membrane swabs were taken during the 60 min of hospitalization due to the characteristic odour of the patient. According to the medical records, blood was drawn within 1 hour of the patient’s admission for the treatment. During hospitalization, the Department of Toxicology (Acute Poisoning Center) was consulted regarding the patient’s condition and the correct preservation of the biological material and treatment procedure. All toxicological analyses—also those of blood samples taken before death—were performed to determine the cause of poisoning after the patient’s death (Table 2).

Dimethylsulphoxide was found at 32 µg/mL concentrations in the blood taken before death and 16 µg/mL in the autopsy blood. In both matrices, a metabolite of DMSO, dimethylsulfone (DMSO2), was confirmed. According to standard procedures to establish a toxicological profile based on the cause of death, screening tests were performed to exclude the presence of ethyl alcohol as well as the psychoactive substances and drugs most commonly identified in acute poisoning, i.e., morphine derivatives, amphetamine, methamphetamine, cocaine, benzodiazepines, barbiturates, tricyclic antidepressants, methadone and cannabinoids. The study was performed using a high-performance liquid chromatography method in a gradient flow mobile phase with detection in the form of tandem mass spectrometry in the scanning mode and multiple reaction monitoring (MRM). In addition, a gas chromatography method coupled to a high-resolution TOF mass spectrometer (time-of-flight analyser, scanning in the range of 30–600 AMU) was used. Blood, urine and skin and mucous membrane swabs were analysed for volatile substances with gas chromatography using the ‘headspace’ technique. Due to its physicochemical properties, detecting hydrogen peroxide in biological material (blood, urine, skin and mucous membrane swabs) was impossible. The only evidence of its involvement in the poisoning was the toxicological tests on the evidence indicating that the concentrations of H_2_O_2_ in the preparations used for the infusions were significantly exceeded.

### 2.4. Trial

Based on the material collected, acute cardiopulmonary failure due to the toxic effects of hydrogen peroxide, administered intravenously as a mechanism for forming air embolisms in the coronary and cerebral vessels, was determined as the cause of death. Due to the content of DMSO in the patient’s blood below toxic concentrations, its contributory role in the patient’s death was excluded. Initially, the investigation conducted in the case concerned all medical professionals involved in the case. After issuing opinions on the cause of the victim’s death, the public prosecutor’s office charged the medical staff (midwife) who prepared and administered the intravenous infusions with manslaughter of the patient. The midwife pleaded guilty to mistakenly preparing the intravenous infusion containing hydrogen peroxide and voluntarily submitted to a sentence of 1.5 years of imprisonment and a 3-year ban on practising as a midwife before the court. In the authorities’ opinion, other medical staff did not contribute to the patient’s death, which was justified by the earlier use of the “therapy” without complications.

## 3. Discussion

DMSO (dimethylsulphoxide) is a colourless, odourless liquid with limited therapeutic use [16,17]. In the 1960s, the first therapeutic use of DMSO was announced, but these trials were halted due to side effects [18]. After several years, research on the therapeutic use of DMSO was resumed [18]. Pleiotropic effects on the metabolism and division of immune cells and their secretion of inflammatory mediators have been postulated [19,20,21,22]. Subsequently, there have been attempts to use DMSO in treating inflammatory diseases and, due to its ability to cross the blood–brain barrier, neurological or psychiatric disorders [19,23,24,25,26,27,28]. Due to its high permeability through biological membranes, the compound has found use as a substance that facilitates the permeation of therapeutic substances in externally applied drugs (in the form of ointments). Its anti-inflammatory effect through its influence on immune cells is only used in rheumatoid arthritis and the palliative treatment of interstitial cystitis. It is now used in medicine and biotechnology as a cryopreservative for cell preparations to prevent the formation of intra- and extracellular ice crystals and to stabilise cell membranes [17,29]. In this application, concentrations in the range of 5–10% are used [29]. DMSO administered intravenously may produce side effects depending on the total dose [16,19,29]. The most common include nausea, vomiting, diarrhoea, headache and halitosis [17,18]. The odour emitting from the mouth after the administration of DMSO is described as sulphurous or garlicky [18]. DMSO concentrations in the 30–60% range are always associated with side effects [17]. According to the literature, the LD_50_ of DMSO (lethal dose for 50% of the study population) for mammals is 10–20 g/kg body weight. Also, due to its high osmolality, rapid DMSO solution administration can result in blood cell haemolysis [17]. In addition, rare cases of cardiovascular (e.g., hypotension, hypertension, bradycardia), respiratory (e.g., cough, pulmonary oedema), neurological (e.g., convulsions, encephalopathy) and urogenital (e.g., dysuria, renal failure) complications are reported in the literature. Many factors influence the presence and severity of side effects. Among those currently postulated, in addition to the total dose, are the age and sex of the patient and the mode of administration of DMSO; intravenous, transdermal, oral or eye-drop administrations have been described. In the presented death case, it is suggested that intravenous administration may have been associated with a side effect of halitosis, noted in the medical records as a strong sulphurous odour. In addition, the cardiovascular and general organ complications accompanying the poisoning are noteworthy. Considering the above data, the levels of DMSO found in both before-death and post-mortem blood were below toxic concentrations. In addition, the dimethylsulphoxide metabolite dimethylsulfone (methylsulfonylmethane) was confirmed, which according to the literature, is not a toxic substance. There are no data to indicate its toxicity.

In the case of hydrogen peroxide, there is no medical rationale for using intravenous infusion. Upon contact with catalase, this substance decomposes into water and gaseous oxygen, which contributes to oxidative stress damage and the formation of gas bubbles, causing congestion [30,31]. Just 10 mL of 6% hydrogen peroxide gives off 200 mL of gas, and 100 mL of a 35% solution produces 12–14 L of gas [30,32]. The solution’s oral ingestion often results in acute hydrogen peroxide poisoning [30]. This most commonly affects the elderly or children [30,33,34]. The topical application of a 10% solution to mucous membranes (e.g., oral cavity or gastrointestinal tract) causes tissue damage, and higher-concentration solutions can additionally cause fatal cardiopulmonary failure [30,35,36,37]. Therefore, hyperbaric oxygen treatment is used for hydrogen peroxide poisoning [30,38,39]. The literature describes some complications from excess gas release associated with using hydrogen peroxide [32,38,40]. After intravenous administration, oxygen bubbles are produced inside the vessels and can cause an air embolism in organs [30,41]. There may be two routes for their formation in the brain: the presence of a patent foramen ovale or the passage of the substance through the pulmonary circulation without the prior release of oxygen [30,32]. Gas bubbles may be visible on imaging, but their absence is not a basis for excluding an air embolism [30]. Air embolisms can also form in the coronary vessels of the heart, causing ischaemia, especially after intravenous administration without the need for an anatomical defect in the heart, which becomes the first cavity where gas can collect. The blockage of coronary vessels with gas bubbles results in limiting the delivery of oxygen and nutrients to the cardiomyocytes, resulting in cardiac arrest. Such a mechanism took place in the described case, which is confirmed by the recorded symptoms and the results of laboratory tests. After oral administration, gas bubbles are often visible in the portal vein and its tributaries, from where they can also migrate to other distant areas of the circulatory system [42].

In addition, each case of drug poisoning requires an interdisciplinary approach. Post-mortem diagnostics at the initial stage requires a thorough analysis of medical records and information about the place and course of the event, which enables the proper selection of diagnostic tools during the autopsy. Close cooperation between the coroner and the forensic toxicologist is also necessary from the early stages of the investigation in order to properly select and collect diagnostic material. This is particularly important in situations where the substances that led to poisoning and their doses are unknown [43,44].

## 4. Conclusions

The lack of accurate information about the intravenous substances given to the patient prevented the implementation of treatment aimed at the effects of intravenous hydrogen peroxide administration. The collected history and observed symptoms, including the characteristic odour, led to a misdiagnosis of a DMSO overdose, which was not the main cause of the symptoms presented by the patient. In addition, using experimental, unsafe and Evidence-Based Medicine (EBM) incompatible therapies in ‘natural medicine clinics’ can pose a real risk to the patient’s health and life. The use of chemicals and reagents not registered as medicines and their preparation by staff with no pharmaceutical experience (in this case, the midwife) is an additional element that endangers the health of those undergoing ‘treatment’ in such clinics. The strict supervision of therapies and agents applied in similar clinics is necessary to avoid similar occurrences in the future.

## Figures and Tables

**Table 1:** Content of DMSO and H_2_O_2_ in evidence.

	Volume of Liquids Secured for Testing (mL)	DMSO Content	H_2_O_2_ Content
No. 1	less than 0.1	71%	---
No. 2	less than 0.1	60%	---
No. 3	7.5	---	2.38%
No. 4	130	---	---
No. 5	5.0	7.8%	---
No. 6	5.5	13%	---
No. 7	160	---	11.05%
No. 8	14	---	4.25%

**Table 2:** The results of toxicological tests on the biological materials.

	DMSO Level (µg/mL)	DMSO2 Presence
blood taken in hospital	32	positive
post-mortem blood	16	positive
urine taken from bladder	<1	positive
skin swab	not found	positive
mucosal swab	not found	positive

## Data Availability

All data supporting the reported results can be found in the archive of the Department of Forensic Medicine of Poznan University of Medical Sciences.
